# Impacts of Constitutive and Induced Benzoxazinoids Levels on Wheat Resistance to the Grain Aphid (*Sitobion avenae*)

**DOI:** 10.3390/metabo11110783

**Published:** 2021-11-16

**Authors:** Zhanfeng Zhang, Hao Lan, Hehe Cao, Xiangshun Hu, Yongliang Fan, Yue Song, Lijuan Wu, Tong-Xian Liu

**Affiliations:** 1 State Key Laboratory of Crop Stress Biology in Arid Areas, Key Laboratory of Northwest Loess Plateau Crop Pest Management of Ministry of Agriculture, College of Plant protection, Northwest A&F University, Yangling 712100, China; zhanfengfwjt@nwafu.edu.cn (Z.Z.); lan@nwafu.edu.cn (H.L.); xiangshun@nwafu.end.cn (X.H.); yfan@nwafu.edu.cn (Y.F.); songyue409@nwafu.edu.cn (Y.S.); wu_lijuan@nwafu.edu.cn (L.W.); 2 College of Plant Health and Medicine, Qingdao Agricultural University, Qingdao 266109, China; caohehe1988@qau.edu.cn

**Keywords:** benzoxazinoids, wheat, *Sitobion avenae*, mass spectrometry, aphid resistance, callose deposition

## Abstract

Benzoxazinoids are important secondary metabolites in gramineae plants and have inhibitory and toxic effects against a wide range of herbivore pests. However, the relationship between benzoxazinoid level and plant resistance to aphids remains controversial. In this study, we investigated the relationship between benzoxazinoids composition and concentration in wheat leaves and the resistance to the grain aphid *Sitobion avenae*. Overall, six benzoxazinoids were detected and identified by mass spectrometry based metabolites profiling, including three lactams, two hydroxamic acids, and one methyl derivative. The constitutive levels of these benzoxazinoids were significantly different among the wheat varieties/lines. However, none of these benzoxazinoids exhibited considerable correlation with aphid resistance. *S. avenae* feeding elevated the level of 2-*O*-*β*-D-glucopyranosyloxy-4,7-dimethoxy-(2H)-1,4-benzoxazin-3(4H)-one (HDMBOA-Glc) and reduced the level of 2-*O*-*β*-D-glucopyranosyloxy-4-hydroxy-7-(2H)-methoxy-1,4-benzoxazin-3(4H)-one (DIMBOA-Glc) in some of the wheat varieties/lines. Moreover, aphid-induced level of DIMBOA-Glc was positively related with callose deposition, which was closely associated with aphid resistance. Wheat leaves infiltrated with DIMBOA-Glc caused a noticeable increase of callose deposition and the effect was in a dose dependent manner. This study suggests that the constitutive level of benzoxazinoids has limited impact on *S. avenae*. Aphid feeding can affect the balance of benzoxazinoids metabolism and the dynamic level of benzoxazinoids can act as a signal of callose deposition for *S. avenae* resistance. This study will extend our understanding of aphid–wheat interaction and provides new insights in aphid-resistance wheat breeding.

## 1. Introduction

Wheat (*Triticum aestivum* L.) is one of the most important crops in the world and crucial to global food security [1]. The grain aphid, *Sitobion avenae* (Fabricius), is a major pest of wheat, and it seriously affects the quality and yield of wheat [2,3]. Currently, chemical pesticide spraying is the main strategy of controlling *S. avenae*, but the side-effects of pesticides have brought more serious challenges for environment and public health [4]. Therefore, growing aphid-resistant wheat varieties is considered as the most economical, safe, and environment-friendly method to control *S. avenae*. However, the lack of aphid resistance germplasm has seriously hindered the process of breeding aphid-resistant wheat varieties [5,6].

Exploring plant secondary metabolites as defense chemicals is an effective way to screen the natural resistant wheat against aphids [7,8,9]. Benzoxazinoids are important defense metabolites in gramineous plants (Poaceae), such as wheat [10]. Benzoxazinoids have been reported to function as antifeedant, insecticidal, antimicrobial, and allelopathic properties [11,12]. Many studies have demonstrated that benzoxazinoids have antifeedant and toxic effects on a wide range of insect herbivores [12]. However, the relationship between benzoxazinoids and aphid resistance remains controversial [13,14,15,16,17,18,19,20,21,22,23]. Some researchers found that the constitutive benzoxazinoid levels in host plants were positively correlated with resistance against various species of aphids, such as *S. avenae*, *Schizaphis graminum*, *Rhopalosiphum padi*, and *R. maidis* [13,14,15,16,17]. Other studies suggested that the concentration and composition of benzoxazinoids were not the primary factor of aphid resistance [18,19,20,21,22]. In some cases, aphids even perform better on plants with higher levels of benzoxazinoids [23]. Benzoxazinoids related plant defense can also be induced upon aphid feeding, depending on aphid species and host plant genetic background [17,21]. However, the relative contributions of constitutive and induced benzoxazinoid levels to aphid resistance remain poorly understood.

Benzoxazinoids are divided into three groups based on their structures as lactams, hydroxamic acids, and methyl derivatives (Table 1) [11,24]. To date, the analysis of benzoxazinoids relies mostly on spectrophotometry, liquid chromatography coupled with ultraviolet detection (LC-UV), gas chromatography coupled with mass spectrometry (GC-MS), and liquid chromatography coupled with mass spectrometry (LC-MS) [25]. Nevertheless, spectrophotometry is vulnerable to interference from other potential impurities and has a poor selectivity. LC-UV is a selective and sensitive analytical technique for separation and quantification of complex mixture. However, one of the major difficulties of the LC-UV technique is scarcity of commercial standard compounds as reference substances [26,27]. Due to low volatility, benzoxazinoids are not suitable for direct GC-MS analysis, and a time-consuming derivatization step is needed before analysis [28]. To overcome these technique limitations, liquid chromatography coupled with tandem multistage MS (LC-MS^n^) have been developed. Compared with single-quadrupole MS, tandem multistage MS (MS^n^) can provide a wealth of structural information and provide more insights into the detailed structure of a target compound [29]. Many kinds of benzoxazinoids have been identified by tandem multistage spectrometry [30,31,32]. In the present study, we used a LC-MS based approach for benzoxazinoids profiling in 13 wheat varieties/lines. In addition, we evaluated the relative contributions of constitutive and induced benzoxazinoid levels to aphid resistance in wheat plants. This study extends our understanding of the biological roles of benzoxazinoids in aphid–wheat interaction and provides new insights in aphid-resistance wheat breeding.

## 2. Results

### 2.1. Evaluation of Wheat Resistance to S. avenae

**Figure 1:**
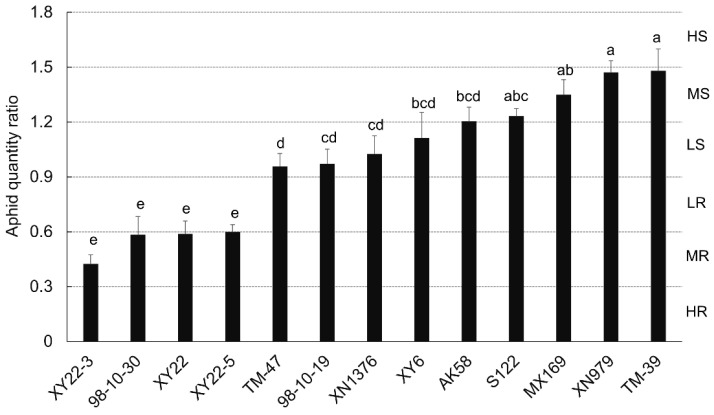
The degree of resistance to *Sitobion avenae* in 13 wheat varieties/lines. The degrees of resistance were evaluated by aphid quantity ratio (AQR) method. XY6 stands for the variety of Xiaoyan6; XY22 stands for the variety of Xiaoyan22; XY22-3 and XY22-5 are the sib-lines of XY22; TM-39 stands for the line of 186Tm39; TM-47 stands for the line of 186Tm47; XN979 stands for the variety of Xinong979; MX169 stands for the variety of Mingxian169; AK58 stands for the variety of Aikang58; S122 stands for the variety of Shan122; XN1376 stands for the variety of Xinong1376. HR stands for high resistant; MR stands for median resistant; LR stands for low resistant; LS stands for low susceptible; MS stands for median susceptible; HS stands for high susceptible. Different lowercase letters on the top of the bar indicate significant differences at *p* < 0.05 (Tukey’s HSD test).

The degree of resistance to *S. avenae* in 13 varieties/lines was evaluated by aphid quantity ratio (AQR), which is defined as the number of aphids in each seedling divided by the average number of aphids in all testing seedlings. The results showed that the AQR was significantly affected by the wheat varieties/lines (*F*_12, 65_ = 17.706, *p* < 0.001) (Figure 1). The AQR from the wheat lines XY22-3, 98-10-30, XY22, and XY22-5 were significantly less than other nine varieties/lines and exhibited medial resistance (MR) to *S. avenae*. The AQR from AK58, S122, MX169, XN979, and TM-39 were greater than other eight varieties/lines and exhibited median susceptible (MS). The other four varieties/lines exhibited low susceptible (LS) to *S. avenae*.

### 2.2. Chromatographic and Mass Spectrometric Behavior of Benzoxazinoids

**Figure 2:**
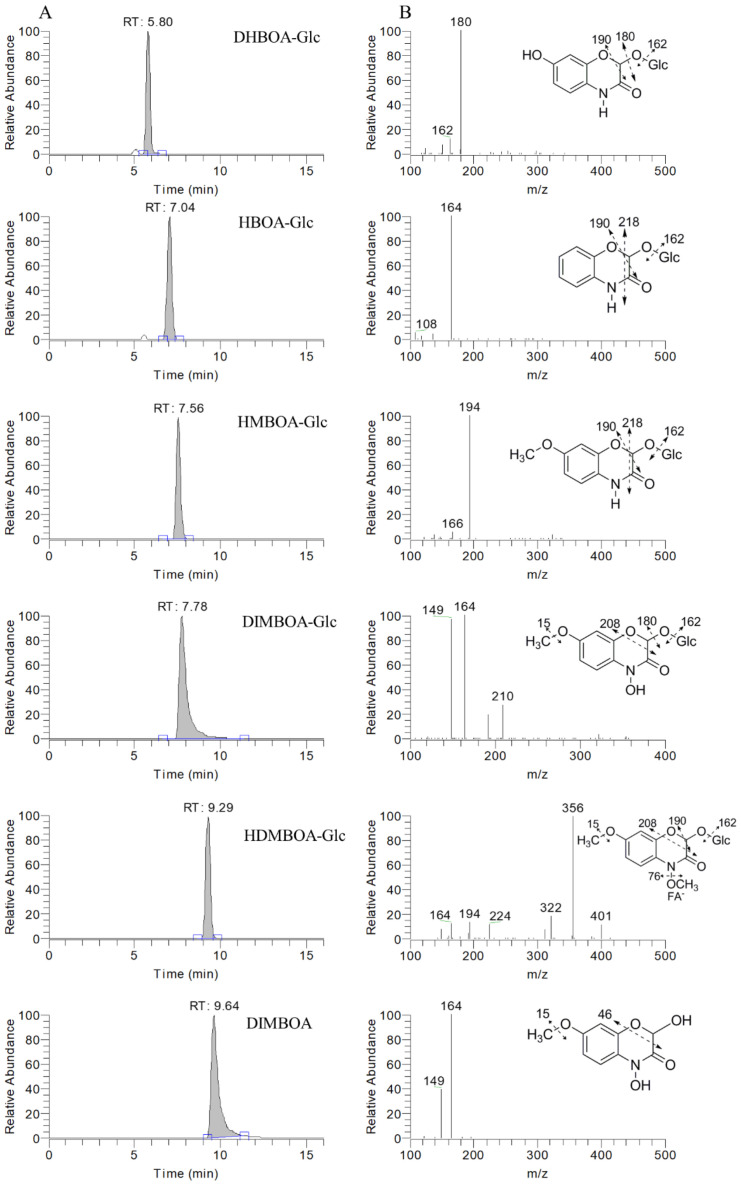
Extracted ion chromatograms, mass spectra, and fragmentation pathways of benzoxazinoids. (**A**) Extracted ion chromatograms. RT, retention time; the blue line indicates the baseline of the peak; the blue squares indicate the start time and end time of the peak. (**B**) Mass spectra and fragmentation pathways of benzoxazinoids. The dotted arrow indicates a putative cleavage with the observed mass fragments. *m*/*z*, mass-to-charge ratio; the number in front of the arrow indicates the molecular weight of the neutral loss fragment.

The optimal chromatographic separation was achieved using a linear gradient with methanol-formic acid aqueous solution and all target compounds were eluted within 10 min (Figure 2A). Different mass spectrometric parameters were optimized for each benzoxazinoid to obtain structural information and to achieve maximum sensitivity. In negative ionization mode, benzoxazinoids had higher sensitivity and more stable multistage mass spectrometric behavior. Six benzoxazinoids were identified based on their mass spectrometry information and confirmed by standard compounds. These compounds were as follows: three lactams: 2-*O-β*-D-glucopyranosyloxy-7-hydroxy-(2H)-1,4-benzoxazin-3(4H)-one (DHBOA-Glc), 2-*O-β*-D-glucopyranosyloxy-1,4-benzoxazin-3(4H)-one (HBOA-Glc), and 2-*O-β*-D-glucopyranosyloxy-7-methoxy-(2H)-1,4-benzoxazin-3(4H)-one (HMBOA-Glc); two hydroxamic acids: 2,4-dihydroxy-7-methoxy-1,4-benzoxazin-3-one (DIMBOA) and 2-*O-β*-D-glucopyranosy-loxy-4-hydroxy-7-(2H)-methoxy-1,4-benzoxazin-3(4H)-one (DIMBOA-Glc); and one methyl derivatives: 2-*O-β*-D-glucopyranosyloxy-4,7-dimethoxy-(2H)-1,4-benzoxazin-3(4H)-one (HDMBOA-Glc). Five out of six benzoxazinoids were identified as glucosides, and only one aglycon (DIMBOA) was detected. Benzoxazinoids usually formed the deprotonated ion [M − H]^−^ and the formic acid adduct [M + FA − H]^−^ in negative full MS mode. Benzoxazinoids from the different subclasses showed typical ionization and multistage fragmentation behaviors. Typical neutral loss fragments of lactams are 162, 180, 190, and 218 Da in MS_2_ spectrum, corresponding to neutral losses of glycan residue (Glc), Glc + H_2_O, Glc + CO, and Glc + 2CO, respectively. Fragmentation of hydroxamic acid typically formed fragments at mass-to-charge ratio of 210, 164, and 149, corresponding to neutral losses of glycan residue (162 Da), CH_2_O_2_ (46 Da), and C_2_H_5_O_2_ (61 Da), respectively. Ionization behavior of methyl derivatives was different from other benzoxazinoids. The molecular ion of methyl derivatives was formic acid adduct [M + FA − H]^−^ and the deprotonated ion was not detected. Typical neutral loss fragments of methyl derivatives were as follows: OCH_3_ (31 Da), FA + OCH_3_ (76 Da), Glc + CH_2_O_2_ (208 Da), FA + Glc + OCH_3_ (238 Da), FA + Glc + C_2_H_5_O_2_ (268 Da), and FA + Glc + C_3_H_8_O_2_ (283 Da). The putative fragmentation pathways of detected benzoxazinoids and detailed mass spectra information are shown in Figure 2B and Table S1.

### 2.3. Constitutive Benzoxazinoid Levels in Wheat Seedlings and Their Correlation with S. avenae Resistance

**Figure 3:**
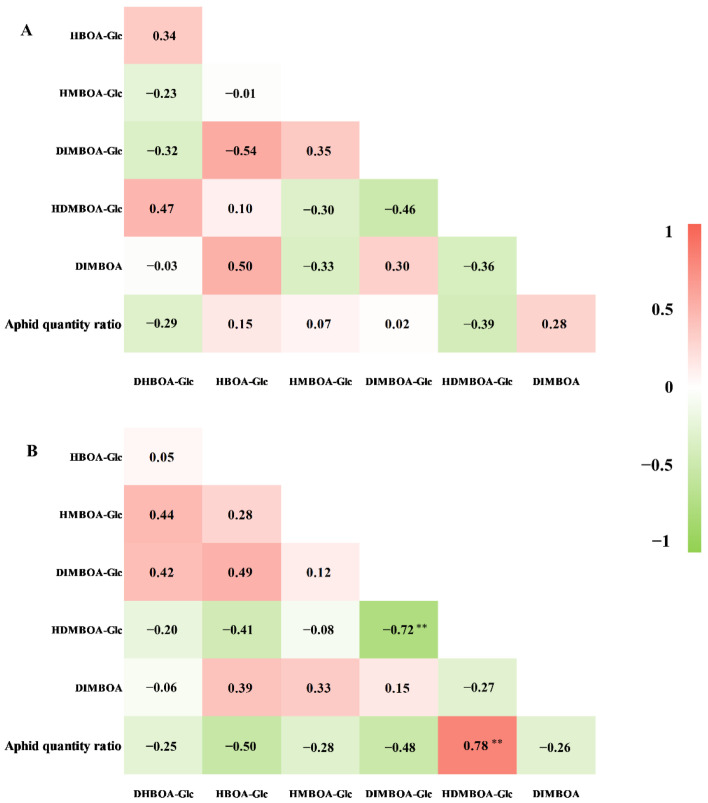
Multivariate correlation between benzoxazinoid levels and aphid resistance. (**A**) Constitutive benzoxazinoid levels; (**B**) induced benzoxazinoid levels; ** indicates significant correlation at the *p* < 0.01 level.

As shown in Table 2, the constitutive benzoxazinoid levels in wheat seedlings varied markedly among the 13 wheat varieties/lines, even in the sib-lines with similar genetic background, such as XY22, XY22-3, and XY22-5. By contrast, DIMBOA-Glc was the major benzoxazinoid in wheat leaves and the minimum and maximum levels were 275.77 and 901.01 μg/g fresh weight (FW) for XY22-5 and S122, respectively. DIMBOA was found at the lowest content in all wheat varieties/lines, and the content ranged from 4.00 to 34.06 μg/g FW for MX169 and AK58, respectively. Correlation analysis showed that the constitutive benzoxazinoid levels were not correlated with resistance level to *S. avenae* (Figure 3A).

### 2.4. Induced Levels of Benzoxazinoids in Aphid Infested Wheat Seedlings and Their Correlation with S. avenae Resistance

**Figure 4:**
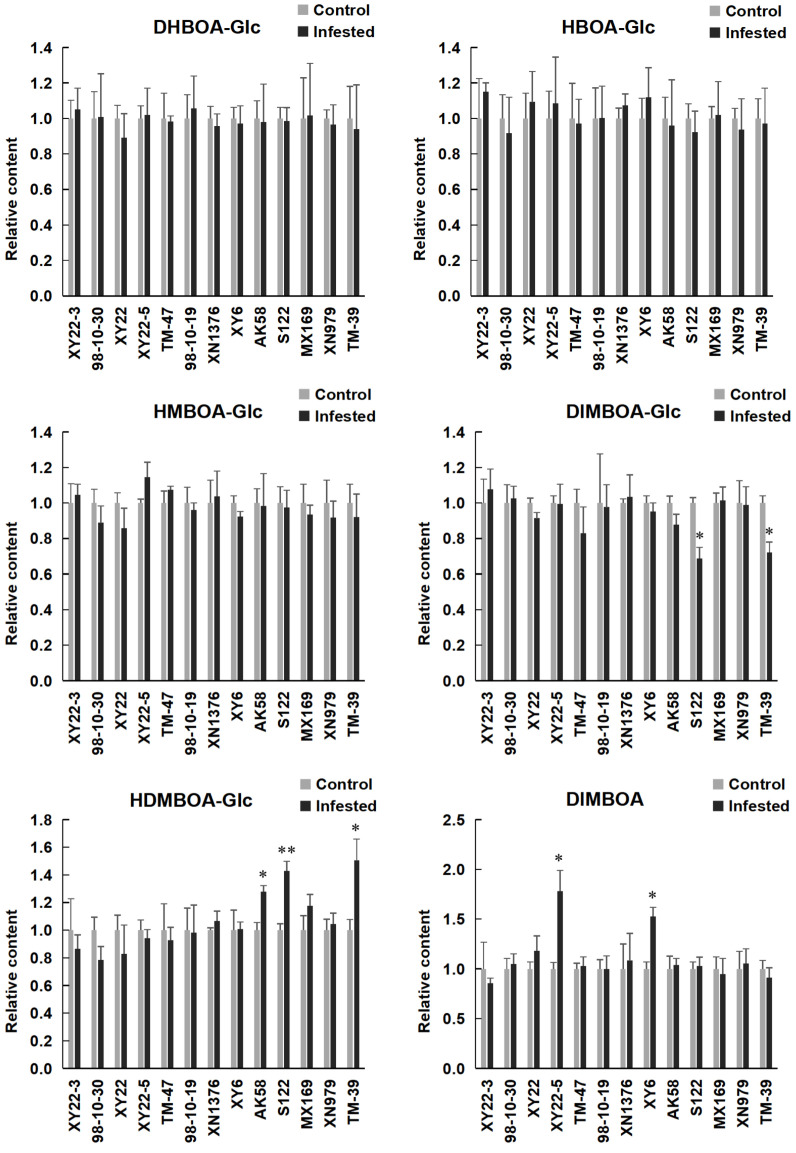
Relative amount of benzoxazinoid contents in the control and aphid infested wheat leaves. Mean values were normalized to the mean of control wheat seedlings with no aphids. Stars indicate significant differences calculated by Student’s *t*-test: * *p* < 0.05; ** *p* < 0.01.

The induced levels of the benzoxazinoids were measured by the peak areas of main ions of their corresponding MS_2_ spectra. Mean values were normalized to the mean of control wheat seedlings with no aphids (Figure 4). *S*. *avenae* feeding altered several benzoxazinoid levels in some of the wheat varieties/lines. Specifically, the levels of DIMBOA were significantly increased in XY6 and XY22-5 after 96 h of *S. avenae* feeding. The levels of DIMBOA-Glc were significantly decreased in TM-39 and S122, while the levels of HDMBOA-Glc were significantly increased in TM-39, S122, and AK58 after *S. avenae* feeding. A significant negative correlation was found between the relative abundance of HDMBOA-Glc and DIMBOA-Glc (*r* = −0.72, *p* < 0.01, Figure 3B). Furthermore, the levels of DHBOA-Glc, HBOA-Glc, and HMBOA-Glc were not affected by *S. avenae* feeding. Correlation analysis of the benzoxazinoid levels showed obvious differences between control and aphid infested wheat seedlings (Figure 3B). A positive linear correlation was found between the relative abundance of HDMBOA-Glc and AQR in aphid infested wheat seedlings, while there was no clear correlation between the induced level of other benzoxazinoids and *S. avenae* resistance.

### 2.5. The Relationship between Aphid-Induced Benzoxazinoids Levels and Callose Deposition

**Figure 5:**
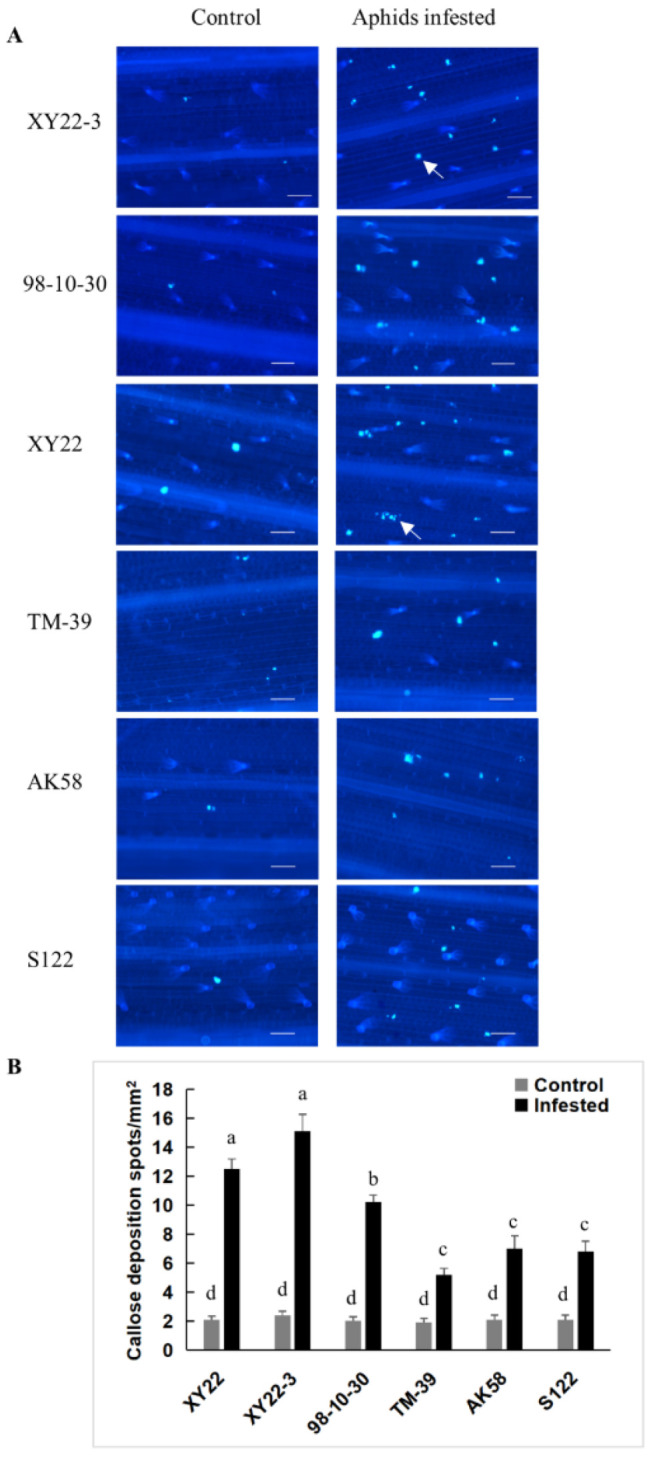
Aphid-induced callose deposition in different wheat varieties/lines. (**A**) Histochemical staining of callose in control and *S. avenae* infested wheat leaves. Callose was stained with aniline blue and visualized as bright-blue spots (indicated by white arrows). Scale bar = 100 µm. (**B**) Quantification of callose deposition spots in control and *S. avenae* infested wheat leaves. Different letters indicate significant differences (Kruskal–Wallis test).

**Figure 6:**
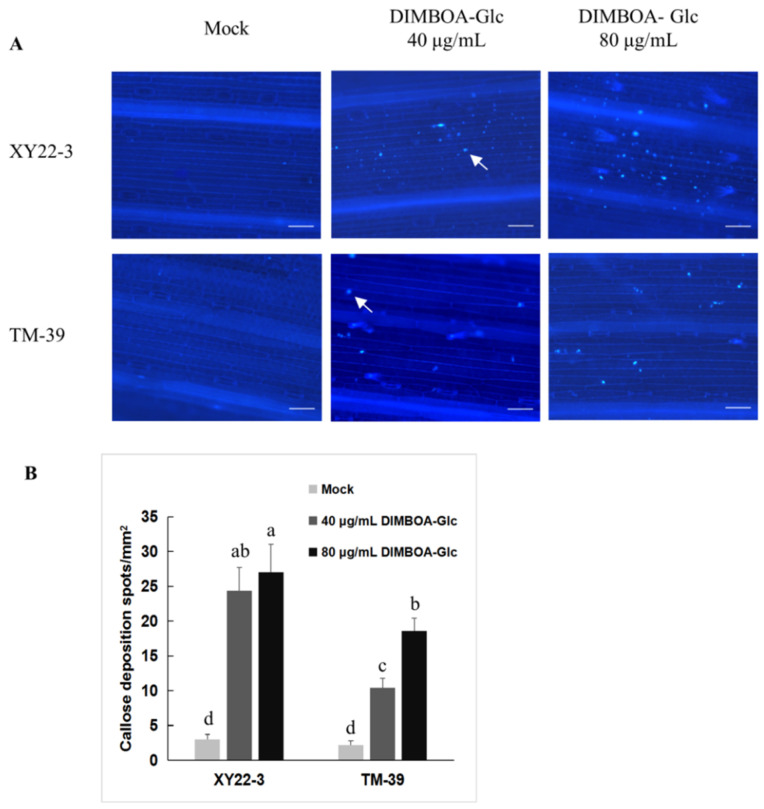
DIMBOA-Glc induced callose deposition in wheat leaves. (**A**) Histochemical staining of callose in wheat leaves infiltrated with different concentrations of DIMBOA-Glc. Callose was visualized as bright-blue spots by staining with aniline blue (indicated by white arrows). Scale bar = 100 µm. (**B**) Quantification of callose deposition spots in wheat leaves infiltrated with DIMBOA-Glc. Different letters indicate statistically significant differences (ANOVA, followed by Tukey’s HSD test).

To investigate the relationship between benzoxazinoids levels and callose deposition, we tested callose deposition in control and aphid infested wheat seedlings. As illustrated in Figure 5, callose deposition was detected in the leaf epidermis of all tested plants. There were few callose deposition spots in control seedlings and the number of callose deposition spots did not differ significantly among control plants. *S. avenae* feeding caused a noticeable increase in the number of callose deposition spots, especially in aphid-resistant wheat leaves, such as XY22-3, 98-10-30, and XY22 (Figure 5). In the epidermis of aphid infested leaves, the number of aphid-induced callose deposition spots was significantly lower in aphid-susceptible seedlings than in aphid-resistant seedlings, which also showed reduced DIMBOA-Glc levels and elevated HDMBOA-Glc levels. Correlation analysis showed the number of aphid-induced callose deposition spots had a positive relation with DIMBOA-Glc levels (*r* = 0.84, *p* < 0.05), while had a negative relation with HDMBOA-Glc (*r* = −0.87, *p* < 0.05) levels. Wheat leaves infiltrated with DIMBOA-Glc induced significant callose deposition, especially in aphid-resistant wheat line XY22-3. Infiltration with 40 or 80 μg/mL DIMBOA-Glc in XY22-3 elicited more callose deposition spots than in TM-39. The effect of DIMBOA-Glc was in a dose dependent manner in wheat line TM-39 (Figure 6).

## 3. Discussion

Benzoxazinoids are synthesized by indole-3-glycerol phosphate in the plastids. Normally, toxic and unstable benzoxazinoid-aglycones are glycosylated and stored as benzoxazinoid glucosides in vacuoles to prevent self-toxicity in undamaged plant cells [10,11]. Benzoxazinoid glucosides are vulnerable to hydrolysis by β-glucosidase during plant tissue disruption, and thus the extraction and quantitative analysis of benzoxazinoids in plants were quite challenging [25]. In our present work, wheat samples were frozen and ground into powder in liquid nitrogen immediately after sample collecting. Furthermore, the subsequent extraction was carried out at low temperature to prevent hydrolysis of benzoxazinoids by β-glucosidase. In this study, five out of six benzoxazinoids were identified as glucosides and only one aglucone (DIMBOA) was detected in wheat leaves. There was no correlation between the contents of DIMBOA and its glucoside compound, DIMBOA-Glc. Similar to previous studies, DIMBOA has been found in wheat tissue when β-glucosidase activity was restricted by liquid nitrogen [9,33,34]. These results indicate that DIMBOA is synthesized constitutively in wheat leaves.

Benzoxazinoid composition and abundance largely depend on plant species, cultivar, developmental stages, and environmental factors [24]. For example, the major benzoxazinoid in rye is DIBOA-Glc, whereas the most abundant benzoxazinoid in maize is DIMBOA-Glc [35,36]. In wheat plants, DIMBOA-Glc, HMBOA-Glc, HDMBOA-Glc, and DIMBOA are the most frequently identified benzoxazinoids [9,21,22,32,33,34]. Including these four benzoxazinoids, a total of six benzoxazinoids were identified in this study and the most abundant benzoxazinoid was DIMBOA-Glc. The constitutive levels of these benzoxazinoids differed widely among different wheat varieties/lines, even in the sib-lines with similar genetic background, such as XY22, XY22-3, and XY22-5. Previous studies also indicated that levels of benzoxazinoids varied greatly in different wheat varieties/lines of the same developmental stage [21,22,37].

Benzoxazinoids can disturb cell function by inhibition of many target enzymes [11]. The resistance of benzoxazinoid towards herbivores can be implemented by toxic and antifeedant activities. Benzoxazinoids have been shown to be toxic to many chewing herbivores, such as *Ostrinia nubilalis*, *O. furnacalis, Spodoptera exigua*, and *S. frugiperda* [38,39,40,41]. Compared to chewing herbivores, aphids feed upon phloem sap with their stylet-like mouthparts from plant sieve elements and cause little damage to host plants [42]. The effects of benzoxazinoids on aphids have been studied using both artificial diets and plants containing different levels of benzoxazinoids. In artificial diet experiments, most of the benzoxazinoids exhibited negative effects on a wide range of aphid species, such as *S. avenae* [16], *R. padi* [17], *Metopolophium dirhodium* [43], and *S. graminum* [44]. However, previous studies showed inconsistent results in aphid performance assay with different host plants. Some studies showed that the constitutive benzoxazinoid levels in host plants are positively correlated with resistance to cereal aphids [13,14,15,45], while some other researchers found the concentration and composition of benzoxazinoids were not correlated with aphid resistance [18,19,20,21,22,23]. In this study, although the *S. avenae* resistance level was significantly affected by wheat varieties/lines, the constitutive levels of benzoxazinoids were not correlated with *S. avenae* resistance level. Our results suggested that the constitutive level of benzoxazinoids is not the primary factor responsible for wheat resistance to *S. avenae* in these wheat varieties/lines. Typically, benzoxazinoids are synthesized in plastids and mobilized in the phloem sap and their effect on aphids depends on their abundance in the phloem. According to previous studies, benzoxazinoid levels in the phloem sap are much lower than that of the whole wheat leaves and aphids can avoid the higher toxicity levels of benzoxazinoids in mesophyll cells by careful probing [34,46]. Moreover, aphids can avoid the negative effects of benzoxazinoids due to the detoxification system [23]. Therefore, the direct toxicity of benzoxazinoids has a limited effect on *S. avenae* in wheat.

In this study, we found that *S. avenae* feeding had different impact on benzoxazinoid levels in wheat plants. Specifically, the level of DIMBOA was significantly increased in some wheat varieties/lines, while DIMBOA-Glc level was decreased after *S. avenae* feeding. Our results are consistent with previous studies that aphid feeding led to the hydrolysis of DIMBOA-Glc and resulted in an increased level of DIMBOA in wheat plants [21,34]. *S. avenae* feeding also altered the level of HDMBOA-Glc, a methyl derivative of DIMBOA-Glc. Previous studies have similarly confirmed that the level of HDMBOA-Glc over-accumulated due to aphids feeding in durum wheat and emmer wheat [34,47]. In addition, we also found that some of the benzoxazinoids (e.g., DHBOA-Glc, HBOA-Glc, and HMBOA-Glc) were not affected by *S. avenae* feeding. Similar phenomena were also observed in maize and wheat plants, respectively [48,49,50]. Overall, these findings indicate that the induced benzoxazinoid level is affected by the properties of benzoxazinoids and plant genotypes.

Apart from direct toxicity of benzoxazinoids, some of the benzoxazinoids, such as DIMBOA-Glc, can act as signal molecules and induce callose deposition for aphid resistance [10,12]. Callose deposition can hinder aphid stylets’ access to the phloem by making cell walls more difficult to penetrate. Callose deposition can also block sieve elements to limit nutrient loss of host plants [12]. Callose deposition has been demonstrated to be an important plant defense mechanism in response to aphid feeding [10,11,12]. In this study, we found that *S*. *avenae* feeding caused a noticeable increase in the number of callose depositions. The number of aphid-induced callose deposition had a positive relation with aphid-induced DIMBOA-Glc level, while had a negative relation with HDMBOA-Glc level in some of the wheat varieties/lines. Furthermore, wheat leaves infiltrated with DIMBOA-Glc significant induced callose deposition and this effect was in a dose dependent manner. These results suggest that aphid-induced DIMBOA-Glc level may have a positive effect on callose deposition in wheat plants, while the induced level of HDMBOA-Glc has an opposite effect. Similarly, a previous study showed that *R. maidis* performed better on maize inbred lines with low DIMBOA-Glc content and high HDMBOA-Glc content, although HDMBOA-Glc was more toxic to *R. maidis* than DIMBOA-Glc [13]. In wheat plants, *S. avenae* grew better on DIMBOA-Glc O-methyltransferases transgenic plants, which exhibited high HDMBOA-Glc level and very low DIMBOA-Glc level [50]. These results suggested that DIMBOA-Glc metabolism had a significant impact on aphid-induced callose deposition. Converting DIMBOA-Glc to HDMBOA-Glc reduced the DIMBOA-Glc level and subsequently suppressed callose deposition and aphid resistance. In addition, the aglucone of DIMBOA-Glc, DIMBOA also exhibited the inducibility of callose in previous studies [17,50]. However, neither constitutive nor induced DIMBOA level exhibited a considerable correlation with callose deposition in this study. According to our results, DIMBOA level was much lower compared with DIMBOA-Glc, so the callose inducibility of DIMBOA may be limited in wheat. Although callose depositions induced by benzoxazinoids in wheat are well established, the detailed mechanism remains poorly understood. Further studies are still needed to clarify the genetic basis and molecular mechanisms of benzoxazinoids dependent callose deposition.

## 4. Materials and Methods

### 4.1. Wheat and Aphids

Thirteen winter wheat varieties/lines used in this study are listed in Table S2. All of them are hexaploid wheat with stable heredity. Four of the 13 varieties/lines, Xiaoyan6 (XY6), Xiaoyan22 (XY22), Xiaoyan22-3 (XY22-3), and Xiaoyan22-5 (XY22-5), have the introduced chromosomes from *Thinopyrum ponticum*. XY22-3 and XY22-5 are the sib-lines of XY22. Two varieties/lines, 186Tm39 (TM-39) and 186Tm47 (TM-47), are hybrids of *T. aestivum* and *T. monococcum*. Two other varieties/lines, 98-10-19 and 98-10-30 are hybrids of *T*. *aestivum* and *T. turgidum*. Five wheat varieties, Xinong979 (XN979), Mingxian169 (MX169), Aikang58 (AK58), Shan122 (S122), and Xinong1376 (XN1376), are bred by hexaploid wheat. AK58 is a widely cultivated wheat variety in China and was bred by Henan Institute of Science and Technology (Xinxiang, China). MX169 is a traditional wheat variety from Shanxi Agricultural University (Taigu, China). The other 11 wheat varieties/lines (four commercial varieties: XY6, XY22, XN979, and XN1376 and seven breeding lines: XY22-3, XY22-5, TM-39, TM-47, 98-10-19, 98-10-30, and S122) were bred by Northwest A&F University (Yangling, China). All the wheat seeds used in this study were collected and provided by the State Key Laboratory of Crop Stress Biology in Arid Areas (Yangling, China). The aphid-resistance levels of some varieties/lines were evaluated in our previous works [51,52,53]. *S*. *avenae* was originally collected from a wheat field (34°297′ N, 108°071′ E) in Yangling, Shaanxi, China. Aphids were reared in a separate cage on wheat seedlings (var. ‘AK58’) in a climate chamber.

### 4.2. Evaluation of Wheat Resistance to S. avenae

Wheat seeds were germinated at room temperature for 24 h in the dark. Then, one germinated seed was planted in a plastic pot (250 mL) containing a 3:1 mixture of peat moss (Pindstrup Mosebrug A/S; Ryomgaard, Denmark) and vermiculite. All seedlings were maintained in a walk-in growth chamber under the following conditions: 22 °C/18 °C, RH 60 ± 5%, and photoperiod 16:8 h (L:D). Each seedling was watered as needed and covered with a ventilated transparent plastic cylinder (8 cm in diameter and 30 cm in height). Seven day old seedlings were used in this experiment. Three adult aphids were introduced to the first fully expanded leaf of wheat seedlings. The adult aphids were removed after 24 h, and 10 newborn nymphs were left on each seedling. After another five days, the nymphs with winged buds were removed, and five wingless aphids were selected and reared on each seedling. Total number of aphids on each wheat seedling was recorded after 2 weeks. Six replicates were conducted for each variety/line.

The degree of resistance to *S. avenae* in different wheat varieties/lines was evaluated by the ratio of aphid quantity (AQR), according to the previous studies [2,54]. AQR was defined as the number of aphids in each seedling divided by the average number of aphids in all testing seedlings. The *S. avenae* resistance level of each plant was graded as follows: high resistance (HR, AQR ≤ 0.3), middle resistance (MR, 0.3 < AQR ≤ 0.6), low resistance (LR, 0.6 < AQR ≤ 0.9), low susceptible (LS, 0.9 < AQR ≤ 1.2), middle susceptible (MS, 1.2 < AQR ≤ 1.5), and high susceptible (HS, AQR > 1.5), respectively.

*Sitobion avenae* infestation experiment was performed as follows: 7-day-old wheat seedlings were infested with *S. avenae* by placing 10 apterous adult aphids on the first fully expanded leaf, which was covered with a ventilated transparent plastic cylinder (8 cm in diameter and 30 cm in height). After feeding for 96 h, all aphids were removed using a hairbrush and the treated wheat seedlings were used for further experiments. Plants without aphids were used as controls and covered with ventilated transparent plastic cylinder. Wheat leaves from control and aphid-infested plants were rapidly harvested and immediately ground into powder in liquid nitrogen for metabolic analysis.

### 4.3. Benzoxazinoids Analysis

Benzoxazinoids were extracted following a previously published protocol with minor modifications [48]. Fifty milligram wheat leaves were ground by a tissue grinder in liquid nitrogen and extracted for 5 min at 4 °C in 1 mL extraction solvent (methanol/water/formic acid, 50/49.5/0.5, *v*/*v*/*v*). After centrifugation at 13,000× *g* for 5 min, the supernatant was transferred to a sample vial and stored at −80 °C before analysis. Benzoxazinoids were extracted and analyzed by high-performance liquid chromatography electrospray ionization ion trap mass spectrometry (LTQ-XL, Thermo Scientific, Waltham, MA, USA). Liquid chromatography separations were carried out with Xterra^®^ MS C18 column (150  ×  2.1  mm; Waters, Milford, CA, USA) and Intertsil OSD-4 C18 Column (250  ×  3.0  mm; GL Sciences Inc., Tokyo, Japan). Benzoxazinoids were separated with a mobile phase consisting of water and methanol, both of which contained 0.1% formic acid (*v*/*v*). The gradient run was at a flow rate of 0.2 mL/min with 30% methanol for an initial 5 min, methanol concentration which was then increased to 80% in 10 min, and the system was held at 80% methanol for 10 min. The injection volume for all samples was 10 μL and the column temperature was held at 40 °C. The MS parameters were as follows: sheath gas (nitrogen) flow rate, 25 arb; aux gas (nitrogen) flow rate 5 arb; spray voltage 4.5 kV; capillary temperature 275 °C. The MS system worked in the negative electrospray ionization (ESI) mode and helium was used as the collision gas in the ion trap. Data dependent MS^n^ analyses were performed by collision-induced dissociation with normalized collision energy of 35%. Data were acquired and processed using Xcalibur 2.1 software (Thermo Scientific, Waltham, MA, USA). Absolute concentrations of benzoxazinoids were quantified by the commercial standards (J&K Scientific, Beijing, China).

### 4.4. Callose Induction and Visualization

For aphid-induced callose deposition, callose was induced by caging 10 adult aphids on the first leaf for 96 h and plants without aphids were used as controls. Aphid-infested leaf segments were collected from at least 10 different plants per variety/line. Benzoxazinoids infiltration experiment was performed according to a previously published protocol with minor modifications [50]. Briefly, the first leaf from 7-day-old seedlings was cut into 2 cm long pieces and then infiltrated with different concentrations of benzoxazinoids in 1.96% methanol (*v*/*v*) and 0.04% acetic acid (*v*/*v*) aqueous solution for 24 h at room temperature. Five randomly collected leaf segments were conducted for each variety/line. Meanwhile, mock treatments were infiltrated with a solution without benzoxazinoids.

Callose deposition in wheat leaves was visualized by callose staining with aniline blue according to previously published protocols with minor modifications [50]. Briefly, wheat leaves were incubated for 72 h in 95% ethanol until all tissues were transparent. After destaining, wheat leaves were washed 3 times with 0.07 M phosphate buffer (pH = 9.0), and then stained with 0.01% analine blue (*m*/*v*, J&K Scientific, Beijing, China) in 0.07 M phosphate buffer (pH = 9.0) for 4 h. After staining, wheat leaves were rinsed with 0.07 M phosphate buffer (pH = 9.0) 3 times and stored at 4 °C in 0.07 M phosphate buffer (pH = 9.0) until microscopic analysis. Observations were performed with a Nikon 80i fluorescence microscopy (Tokyo, Japan) with UV filter (EX 330–380 nm, BA 420nm). Callose depositions were quantified by calculating the number of callose spots per mm^2^ of wheat leaf.

### 4.5. Statistical Analyses

Statistical tests were performed using the SPSS 23 (SPSS Inc, Chicago, IL, USA). The distribution of the sample was verified by Kolmogorov–Smirnov test and the homogeneity of variance was performed using Levene test. The data of *S. avenae* resistance level were analyzed using analysis of variance (ANOVA). The data of constitutive benzoxazinoid levels were evaluated using one-way ANOVA or Kruskal–Wallis nonparametric test. The differences of benzoxazinoid levels between control and the aphid-infested plants were subjected to two-tailed Student’s *t*-test. The relationship between benzoxazinoid contents and *S. avenae* resistance level was studied by Pearson correlation analysis. The data of callose deposition were evaluated using one-way ANOVA or Kruskal–Wallis nonparametric test. The relationship between aphid-induced benzoxazinoids levels (relative abundance) and callose deposition was studied by Pearson correlation analysis.

## 5. Conclusions

In conclusion, we investigated the relative contributions of constitutive and induced benzoxazinoid levels to *S. avenae* resistance in 13 different wheat varieties/lines. Compared with constitutive level of benzoxazinoids, aphid-induced level of benzoxazinoids is more closely related to *S. avenae* resistance. Our data suggest that *S. avenae* feeding can affect the balance of benzoxazinoids metabolism in wheat plants. The dynamic level of benzoxazinoids can act as signal of callose deposition for *S. avenae* resistance. This study extends our understanding of aphid–wheat interaction and provides new insights in aphid-resistance wheat breeding.

## Figures and Tables

**Table 1:** Classification of benzoxazinoids based on previous studies [11,24,32].

Category	Molecular Structure	Substituent Group			Acronym	MW
		R_1_	R_2_	R_3_		
Lactams	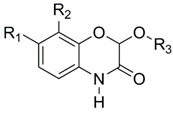	H	H	H	HBOA	165
		H	H	Glc	HBOA-Glc	327
		CH_3_O	H	H	HMBOA	195
		CH_3_O	H	Glc	HMBOA-Glc	357
		CH_3_O	CH_3_O	H	HM_2_BOA	225
		CH_3_O	CH_3_O	Glc	HM_2_BOA-Glc	387
		OH	H	H	DHBOA	181
		OH	H	Glc	DHBOA-Glc	343
Hydroxamic acids	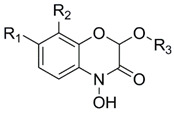	H	H	H	DIBOA	181
		H	H	Glc	DIBOA-Glc	343
		CH_3_O	H	H	DIMBOA	211
		CH_3_O	H	Glc	DIMBOA-Glc	373
		CH_3_O	CH_3_O	H	DIM_2_BOA	241
		CH_3_O	CH_3_O	Glc	DIM_2_BOA-Glc	403
		OH	H	H	TRIBOA	197
		OH	H	Glc	TRIBOA-Glc	359
Methyl derivatives	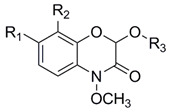	CH_3_O	H	H	HDMBOA	225
		CH_3_O	H	Glc	HDMBOA-Glc	387
		CH_3_O	CH_3_O	H	HDM_2_BOA	255
		CH_3_O	CH_3_O	Glc	HDM_2_BOA-Glc	417

Note: The data were adapted from literatures [11,24,32]; Glc, glucoside; MW, molecular weight.

**Table 2:** Constitutive benzoxazinoid levels (μg/g fresh weight) in the seedling leaves of 13 wheat varieties/lines.

Varieties/Lines	DHBOA-Glc	HBOA-Glc	HMBOA-Glc	DIMBOA-Glc	HDMBOA-Glc	DIMBOA
XY6	91.84 ± 5.72 ^abcd^	433.01 ± 48.94 ^bc^	265.60 ± 10.74 ^ab^	699.55 ± 28.42 ^ab^	67.10 ± 9.72 ^bcd^	7.99 ± 0.56 ^def^
XY22	129.50 ± 9.65 ^ab^	369.41 ± 52.84 ^bcd^	284.19 ± 16.11 ^ab^	669.88 ± 17.93 ^abc^	14.17 ± 1.54 ^g^	7.91 ± 0.56 ^def^
XY22-3	65.36 ± 6.68 ^cde^	250.58 ± 56.20 ^de^	256.60 ± 27.58 ^ab^	754.22 ± 100.70 ^ab^	28.38 ± 6.46 ^defg^	7.58 ± 2.02 ^def^
XY22-5	164.01 ± 11.69 ^a^	379.32 ± 58.29 ^bcd^	239.75 ± 5.21 ^ab^	275.77 ± 11.24 ^e^	140.39 ± 10.26 ^ab^	12.07 ± 0.76 ^cdef^
98-10-19	87.83 ± 11.80 ^abcd^	242.04 ± 41.59 ^de^	220.48 ± 19.50 ^b^	410.60 ± 113.04 ^cde^	43.20 ± 6.88 ^cdef^	13.38 ± 1.24 ^bcd^
98-10-30	106.89 ± 16.12 ^abc^	475.30 ± 63.57 ^ab^	151.67 ± 11.71 ^c^	552.50 ± 56.70 ^bcd^	157.03 ± 14.73 ^a^	6.15 ± 0.64 ^ef^
TM-39	136.84 ± 24.73 ^ab^	602.67 ± 66.70 ^a^	230.48 ± 24.50 ^ab^	699.71 ± 28.45 ^ab^	22.33 ± 1.72 ^fg^	18.11 ± 1.52 ^abc^
TM-47	117.45 ± 16.68 ^abc^	160.89 ± 31.87 ^e^	131.01 ± 8.78 ^c^	283.50 ± 21.64 ^de^	48.93 ± 9.35 ^bcde^	11.61 ± 0.65 ^cde^
XN979	96.75 ± 4.59 ^abc^	293.99 ± 16.55 ^cde^	225.52 ± 29.07 ^ab^	423.55 ± 53.36 ^cde^	63.03 ± 4.97 ^abc^	6.32 ± 1.12 ^def^
MX169	33.27 ± 7.63 ^e^	150.85 ± 10.12 ^e^	284.40 ± 29.78 ^ab^	309.70 ± 17.33 ^de^	37.64 ± 3.93 ^cdef^	4.00 ± 0.48 ^f^
AK58	67.01 ± 6.62 ^bcde^	471.57 ± 56.31 ^ab^	150.20 ± 11.86 ^c^	687.25 ± 26.53 ^ab^	9.10 ± 0.50 ^h^	34.06 ± 4.36 ^a^
XN1376	60.06 ± 4.12 ^de^	260.46 ± 15.07 ^de^	251.27 ± 32.14 ^ab^	806.76 ± 18.01 ^ab^	22.60 ± 0.39 ^efg^	9.05 ± 2.26 ^cdef^
S122	61.65 ± 3.86 ^de^	502.18 ± 40.98 ^ab^	297.49 ± 27.03 ^a^	901.01 ± 27.48 ^a^	23.84 ± 1.11 ^efg^	19.15 ± 1.35 ^ab^

Notes: Values expressed as mean ± SE; means in the same column followed by different lowercase letters are significantly different at *p* < 0.05; comparison of HBOA-Glc and HMBOA-Glc using Tukey HSD test; comparison of DHBOA-Glc, DIMBOA-Glc, HDMBOA-Glc, and DIMBOA using Kruskal–Wallis test.

## Data Availability

The data are available in the article and Supplementary Materials.

## Supplementary Materials

The following are available online at https://www.mdpi.com/article/10.3390/metabo11110783/s1, Table S1: Mass spectra information of benzoxazinoids, Table S2: The wheat varieties/lines used in this study, Table S3: The original data of aphid number and aphid quantity ratio (AQR) in 13 wheat varieties, Table S4: Extracted ion chromatograms and mass spectra of samples, Table S5: The original data of quantitative results of benzoxazinoids in control and aphid infested wheat leaves, Table S6: The original data of callose deposition in control and aphid infested wheat leaves, Table S7: The original data of DIMBOA-Glc induced callose deposition in wheat leaves.

## References

[1] Shewry P.R., Hey S.J. (2015). The contribution of wheat to human diet and health. Food Energy Secur..

[2] Xu Z.H., Chen J.L., Cheng D.F., Sun J.R., Liu Y., Francis F. (2011). Discovery of English grain aphid (Hemiptera: Aphididae) biotypes in China. J. Econ. Entomol..

[3] Wang D., Zhai Y., Liu D., Zhang N., Li C., Shi X. (2019). Identification and genetic differentiation of *Sitobion avenae* (Hemiptera: Aphididae) biotypes in China. J. Econ. Entomol..

[4] Li Y., Hallerman E.M., Wu K., Peng Y. (2020). Insect-Resistant genetically engineered crops in china: Development, application, and prospects for use. Annu. Rev. Entomol..

[5] Aradottir G.I., Martin J.L., Clark S.J., Pickett J.A., Smart L.E. (2017). Searching for wheat resistance to aphids and wheat bulb fly in the historical Watkins and Gediflux wheat collections. Ann. Appl. Biol..

[6] Leonardo C., Singh R.P., Matthew R., Julio H. (2019). Genetics of greenbug resistance in synthetic hexaploid wheat derived germplasm. Front. Plant Sci..

[7] Mithöfer A., Boland W. (2012). Plant defense against herbivores: Chemical aspects. Annu. Rev. Plant Biol..

[8] Yactayo-Chang J.P., Tang H.V., Mendoza J., Christensen S.A., Block A.K. (2020). Plant defense chemicals against insect pests. Agronomy.

[9] Batyrshina Z.S., Yaakov B., Shavit R., Singh A., Tzin V. (2020). Comparative transcriptomic and metabolic analysis of wild and domesticated wheat genotypes reveals differences in chemical and physical defense responses against aphids. BMC Plant Biol..

[10] Niculaes C., Abramov A., Hannemann L., Frey M. (2018). Plant protection by benzoxazinoids—Recent insights into biosynthesis and function. Agronomy.

[11] Wouters F.C., Blanchette B., Gershenzon J., Vassao D.G. (2016). Plant defense and herbivore counter-defense: Benzoxazinoids and insect herbivores. Phytochem. Rev..

[12] Zhou S., Richter A., Jander G. (2018). Beyond defense: Multiple functions of benzoxazinoids in maize metabolism. Plant Cell Physiol..

[13] Givovich A., Niemeyer H.M. (1995). Comparison of the effect of hydroxamic acids from wheat on five species of cereal aphids. Entomol. Exp. Appl..

[14] Argandoña V.H., Corcuera L.J., Niemeyer H.M., Campbell B.C. (1983). Toxicity and feeding deterrency of hydroxamic acids from Gramineae in synthetic diets against the greenbug, *Schizaphis graminum*. Entomol. Exp. Appl..

[15] Thackray D.J., Wrattent S.D., Edwards P.J., Niemeyer H.M. (1990). Resistance to the aphids *Sitobion avenae* and *Rhopalosiphum padi* in Gramineae in relation to hydroxamic acid levels. Ann. Appl. Biol..

[16] Leszczynski B., Dixon A.F. (1990). Resistance of cereals to aphids: Interaction between hydroxamic acids and the aphid *Sitobion avenae* (Homoptera: Aphididae). Ann. Appl. Biol..

[17] Ahmad S., Veyrat N., Gordon-Weeks R., Zhang Y., Martin J., Smart L., Glauser G., Erb M., Flors V., Frey M. (2011). Benzoxazinoid metabolites regulate innate immunity against aphids and fungi in maize. Plant Physiol..

[18] Bing J.W., Guthrie W.D., Dicke F.F., Obryckp J.J. (1990). Relation of corn leaf aphid (Homoptera: Aphididae) colonization to DIMBOA content in maize inbred lines. J. Econ. Entomol..

[19] Kazemi M.H., Van Emden H.F. (1992). Partial antibiosis to *Rhopalosiphum padi* in wheat and some phytochemical correlations. Ann. Appl. Biol..

[20] Castaneda L.E., Figueroa C.C., Fuentes-Contreras E., Niemeyer H.M., Nespolo R.F. (2009). Energetic costs of detoxification systems in herbivores feeding on chemically defended host plants: A correlational study in the grain aphid, *Sitobion avenae*. J. Exp. Biol..

[21] Elek H., Smart L., Martin J., Ahmad S., Gordon-Weeks R., Welham S., Nádasy M., Pickett J.A., Werner C.P. (2013). The potential of hydroxamic acids in tetraploid and hexaploid wheat varieties as resistance factors against the bird-cherry oat aphid, *Rhopalosiphum padi*. Ann. Appl. Biol..

[22] Pereira J.F., Sarria A.L., Powers S.J., Aradottir G.I., Caulfield J.C., Martin J., Smart L.E., Pickett J.A., Birkett M.A., Pereira P.R. (2017). DIMBOA levels in hexaploid Brazilian wheat are not associated with antibiosis against the cereal aphids *Rhopalosiphum padi* and *Sitobion avenae*. Theor. Exp. Plant Physiol..

[23] Castañeda L.E., Figueroa C.C., Nespolo R.F. (2010). Do insect pests perform better on highly defended plants? Costs and benefits of induced detoxification defences in the aphid *Sitobion avenae*. J. Evolution. Biol..

[24] Niemeyer H.M. (2009). Hydroxamic acids derived from 2-hydroxy-2H-1,4-benzoxazin-3(4H)-one: Key defense chemicals of cereals. J. Agric. Food Chem..

[25] Villagrasa M., Eljarrat E., Barceló D., Barceló D. (2009). Analysis of benzoxazinone derivatives in plant tissues and their degradation products in agricultural soils. Trends Analytic. Chem..

[26] Mayoral A.M., Gutierrez C., Ruaz M.L., Castanera P. (1994). A high performance liquid chromatography method for quantification of diboa, DIMBOA, and MBOA from aqueous extracts of corn and winter cereal plants. J. Liquid Chromatogr. Related Technol..

[27] Stochmal A., Kus J., Martyniuk S., Oleszek W. (2006). Concentration of benzoxazinoids in roots of field-grown wheat (*Triticum aestivum* L.) varieties. J. Agric. Food Chem..

[28] Finney M.M., Danehower D.A., Burton J.D. (2005). Gas chromatographic method for the analysis of allelopathic natural products in rye (*Secale cereale* L.). J. Chromatogr. A.

[29] Conceição R.S., Reis I.M.A., Cerqueira A.P.M., Perez C.J., Junior M.C.D.S., Branco A., Ifa D.R., Botura M.B. (2020). Rapid structural characterisation of benzylisoquinoline and aporphine alkaloids from *Ocotea spixiana* acaricide extract by HPTLC-DESI-MS^n^. Phytochem. Anal..

[30] Gao L., Shen G., Zhang L., Qi J., Zhang C., Ma C., Li J., Wang L., Malook S.U., Wu J. (2019). An efficient system composed of maize protoplast transfection and HPLC-MS for studying the biosynthesis and regulation of maize benzoxazinoids. Plant Methods.

[31] Robert C.A., Zhang X., Machado R.A., Schirmer S., Lori M., Mateo P., Erb M., Gershenzon J. (2017). Sequestration and activation of plant toxins protect the western corn rootworm from enemies at multiple trophic levels. eLife.

[32] de Bruijn W.J.C., Vincken J., Duran K., Gruppen H. (2016). Mass spectrometric characterization of benzoxazinoid glycosides from rhizopus-elicited wheat (*Triticum aestivum*) seedlings. J. Agric. Food Chem..

[33] Søltoft M., Jørgensen L.N., Svensmark B., Fomsgaard I.S. (2008). Benzoxazinoid concentrations show correlation with *Fusarium* head blight resistance in Danish wheat varieties. Biochem. Syst. Ecol..

[34] Singh A., Dilkes B., Sela H., Tzin V. (2021). The effectiveness of physical and chemical defense responses of wild emmer wheat against aphids depends on leaf position and genotype. Front. Plant Sci..

[35] Oikawa A., Ishihara A., Iwamura H. (2002). Induction of HDMBOA-Glc accumulation and DIMBOA-Glc 4-O-methyltransferase by jasmonic acid in poaceous plants. Phytochemistry.

[36] Cambier V., Hance T., de Hoffmann E. (2000). Variation of DIMBOA and related compounds content in relation to the age and plant organ in maize. Phytochemistry.

[37] Kowalska I., Kowalczyk M. (2019). Determination of benzoxazinoids in spring and winter varieties of wheat using ultra-performance liquid chromatography coupled with mass spectrometry. Acta Chromatogr..

[38] Campos F., Atkinson J., Arnason J.T., Philogene B., Morand P., Werstiuk N.H., Timmins G. (1989). Toxicokinetics of 2, 4-dihydroxy-7-methoxy-1, 4-benzoxazin-3-one (DIMBOA) in the European corn borer, *Ostrinia nubilalis* (Hübner). J. Chem. Ecol..

[39] Phuong T.T.T., Yamamoto M., Fujii T., Kojima W., Matsuo T., Ishikawa Y. (2016). Comparison of the ability to catabolize DIMBOA, a maize antibiotic, between *Ostrinia furnacalis* and *Ostrinia scapulalis* (Lepidoptera: Crambidae), with reference to their hybrids. Appl. Entomol. Zool..

[40] Tzin V., Lindsay P.L., Christensen S.A., Meihls L.N., Blue L.B., Jander G. (2015). Genetic mapping shows intraspecific variation and transgressive segregation for caterpillar-induced aphid resistance in maize. Mol. Ecol..

[41] Silva Brandão K.L., Murad N.F., Peruchi A., Martins C.H.Z., Omoto C., Figueira A., Brandão M.M., Trigo J.R. (2021). Transcriptome differential co-expression reveals distinct molecular response of fall-armyworm strains to DIMBOA. Pest Manag. Sci..

[42] Züst T., Agrawal A.A. (2016). Mechanisms and evolution of plant resistance to aphids. Nat. Plants.

[43] Argandoña V.H., Luza J.G., Niemeyer H.M., Corcuera L.J. (1980). Role of hydroxamic acids in the resistance of cereals to aphids. Phytochemistry.

[44] Corcuera L.J., Queirolo C.B., Argandoña V.H. (1985). Effects of 2-β-D-glucosyl-4-hydroxy-7-methoxy-1,4-benzoxazin-3-one on *Schizaphis graminum* (Rondani) (Insecta, Aphididae) feeding on artificial diets. Experientia.

[45] Argandoña V.H., Niemeyer H.M., Corcuera L.J. (1981). Effect of content and distribution of hydroxamic acids in wheat on infestation by the aphid *Schizaphis graminum*. Phytochemistry.

[46] Elek H., Smart L., Ahmad S., Anda A., Werner C., Pickett J. (2014). A comparison of the levels of hydroxamic acids in Aegilops speltoides and a hexaploid wheat and effects on *Rhopalosiphum padi* behaviour and fecundity. Acta Biol. Hung..

[47] Shavit R., Batyrshina Z.S., Dotan N., Tzin V. (2018). Cereal aphids differently affect benzoxazinoid levels in durum wheat. PLoS ONE.

[48] Meihls L.N., Handrick V., Glauser G., Barbier H., Kaur H., Haribal M.M., Lipka A.E., Gershenzon J., Buckler E.S., Erb M. (2013). Natural variation in maize aphid resistance is associated with 2,4-Dihydroxy-7-Methoxy-1,4-Benzoxazin-3-One glucoside methyltransferase activity. Plant Cell.

[49] Song J., Liu H., Zhuang H., Zhao C., Xu Y., Wu S., Qi J., Li J., Hettenhausen C., Wu J. (2017). Transcriptomics and alternative splicing analyses reveal large differences between maize lines B73 and Mo17 in response to aphid *Rhopalosiphum padi* infestation. Front. Plant Sci..

[50] Li B., Förster C., Robert C.A.M., Züst T., Hu L., Machado R.A.R., Berset J., Handrick V., Knauer T., Hensel G. (2018). Convergent evolution of a metabolic switch between aphid and caterpillar resistance in cereals. Sci. Adv..

[51] Hu X.S., Keller M.A., Liu X.F., Hu Z.Q., Zhao H.Y., Liu T.X. (2013). The resistance and correlation analysis to three species of cereal aphids (Hemiptera: Aphididae) on 10 wheat varieties or lines. J. Econ. Entomol..

[52] Cao H.H., Pan M.Z., Liu H.R., Wang S.Y., Liu T.X. (2015). Antibiosis and tolerance but not antixenosis to the grain aphid, *Sitobion avenae* (Hemiptera: Aphididae), are essential mechanisms of resistance in a wheat cultivar. Bull. Entomol. Res..

[53] Hu X.S., Liu Y., Wang Y., Wang Z., Yu X., Wang B., Zhang G., Liu X., Hu Z., Zhao H. (2016). Resistance of wheat accessions to the English grain aphid *Sitobion avenae*. PLoS ONE.

[54] Tu X., Fan Y., Mcneill M., Zhang Z. (2018). Including predator presence in a refined model for assessing resistance of alfalfa cultivar to aphids. J. Integr. Agric..

