# Supplementary material for: Impacts of Constitutive and Induced Benzoxazinoids Levels on Wheat Resistance to the Grain Aphid (Sitobion avenae)
Source: Metabolites. 2021 Nov 16;11(11):783. doi: 10.3390/metabo11110783 (PMC8620460; doi:10.3390/metabo11110783)
Supplement: Supplementary file 1 [file metabolites-11-00783-s001.zip › Table S1 Mass spectra information of benzoxazinoids.pdf]

Table S1. Mass spectra information of benzoxazinoids.

Compound	RT(min)	Ionization	Precursor (*m/z*)	Neutral Loss fragment(Da)	MS^2 Ions *m/z* (relative intensity)
DHBOA-Glc	5.80	[M+FA−H]$^-$	388	FA (46)	342（100）
		[M−H]$^-$	342	Glc (162)	180（100）
				Glc + H_2O (180)	162（6）
				Glc + CO (190)	152（6）
HBOA-Glc	7.04	[M−H]$^-$	326	Glc (162)	164（100）
				Glc + CO (190)	136（6）
				Glc + 2CO (218)	108（6）
HMBOA-Glc	7.56	[M−H]$^-$	356	Glc (162)	194（100）
				Glc + CO (190)	166（5）
				Glc + 2CO (218)	138（5）
DIMBOA-Glc	7.78	[M+FA−H]$^-$	418	FA (46)	372（100）
		[M−H]$^-$	372	Glc (162)	210（27）
				Glc + H_2O (180)	192（18）
				Glc + H_2O + CO (208)	164（100）
				Glc + H_2O + CO + CH_3 (223)	149（95）
HDMBOA-Glc	9.29	[M+FA−H]$^-$	432	OCH_3 (31)	401（13）
				OCH_3 + FA - H (76)	356（100）
				Glc + H_2O + CO (208)	224（10）
				Glc + OCH_3 + FA-H (238)	194（21）
				Glc + OCH_3 + CH_2O + FA - H (268)	164（20）
				Glc + OCH_3 + CH_2O + CH_3 + FA - H (283)	149（10）
DIMBOA	9.64	[M+FA−H]$^-$	256	FA (46)	210（100）
		[M−H]$^-$	210	H_2O + CO (46)	164（100）
				H_2O + CO + CH_3 (61)	149（45）

Note: RT, Retention Time; FA, formic acid; [M + FA − H]$^-$, FA adduct; [M − H]$^-$, deprotonated ion; Glc, glucoside.
