# Supplementary material for: Impacts of Constitutive and Induced Benzoxazinoids Levels on Wheat Resistance to the Grain Aphid (Sitobion avenae)
Source: Metabolites. 2021 Nov 16;11(11):783. doi: 10.3390/metabo11110783 (PMC8620460; doi:10.3390/metabo11110783)
Supplement: Supplementary file 1 [file metabolites-11-00783-s001.zip › Table S2 The wheat varieties and lines used in this study.pdf]

Table S2. The wheat varieties/lines used in this study.

Name (Abbreviation)	Origin
Xiaoyan 6 (XY6)	Hybrid of *Triticum aestivum* and *Thinopyrum ponticum*
Xiaoyan 22 (XY22)	(Xiaoyan 6×775-1)×Xiaoyan 107
Xiaoyan22-3(XY22-3)	Sib-line of Xiaoyan 22
Xiaoyan22-5(XY22-5)	Sib-line of Xiaoyan 22
186Tm47(Tm-47)	Hybrid of *T. aestivum* and *T. monococcum*
186Tm39(Tm-39)	Hybrid of *T. aestivum* and *T. monococcum*
98-10-30	Hybrid of *T. aestivum* and *T. turgidum*
98-10-19	Hybrid of *T. aestivum* and *T. turgidum*
Xinong979 (XN979)	Derived line of Xiaoyan 6
Mingxian169 (MX169)	*T. aestivum* from China
Aikang58 (AK58)	*T. aestivum* from China
Shan122 (S122)	*T. aestivum* from China
Xinong1376 (XN1376)	*T. aestivum* from China
